# 4576 Driving Research: An Interdisciplinary, Vibrant, Engaged Network (DRIVEN)

**DOI:** 10.1017/cts.2020.209

**Published:** 2020-07-29

**Authors:** Rebecca Avery Reamey

**Affiliations:** 1 University of Alabama at Birmingham

## Abstract

OBJECTIVES/GOALS: We focus on the following mission aligned activities centered upon optimizing the culture around inclusion, equity and diversity in the clinical and translational research faculty at UAB:

- To identify, support and promote Diversity, Equity and Inclusion (DEI) faculty award recognition and leadership program participation locally, regionally and nationally
- To identify, support and promote senior faculty representation on DEI-focused regional, national, and international scientific advisory committees of foundation, professional society and federal programs
- To identify opportunities and support the development of competitive DEI-focused foundation, professional society and federal grant applications
- To support the academic advancement, promotion and tenure among DRIVEN community
- To sponsor and convene professional development and social activities for the DRIVEN community

METHODS/STUDY POPULATION: A partnership of the Center for Clinical and Translational Science Training Academy and the Scientific Community of Outcomes Researchers (SCOR), DRIVEN is a multi-faceted solution to enhance workforce diversity by promoting individual and collective professional development, recognition, and advancement to foster an inclusive, equitable, and diverse research workforce. DRIVEN provides a platform, a community, and a common place where individuals can access resources to more easily identify opportunities aligned with their specific research goals, as well as peer and network support at every step along their professional journey. DRIVEN is uniquely aligned to assist investigators with applying for funding through NIH diversity supplements, foundation opportunities, and other national awards. RESULTS/ANTICIPATED RESULTS: DRIVEN provides networking opportunities, information, and writing support for funding opportunities. Since its inception, less than a year ago, we have seen an increase in writing groups, matched investigators with funding opportunities, and provided networking opportunities for mentors and mentees to meet and for peer mentoring to occur. The interest and momentum surrounding DRIVEN both from internal advisory groups and external advisory groups is significant and will only continue with the endorsement of UAB leadership. DRIVEN is expected to be used as a tool for the recruitment and retention of diverse faculty not only within the UAB community but across the CCTS Partner Network thus changing healthcare in the region. DISCUSSION/SIGNIFICANCE OF IMPACT: According to the NIH, research shows that diverse teams who capitalize on innovative ideas and distinct perspectives outperform less diverse teams. Not only is achieving diversity in the biomedical research workforce critical, but providing diverse researchers with access to support and community is competitive necessity.

